# Prévalence de l’obésité et du surpoids en milieu scolaire, oasis de Tafilalet, sud-est du Maroc

**DOI:** 10.11604/pamj.2020.35.40.17650

**Published:** 2020-02-12

**Authors:** Karima Azekour, Ilham Idir, Nadia Lahrach, Bachir El Bouhali

**Affiliations:** 1 Département de Biologie, Facultés des Sciences et Techniques, Université Moulay Ismail, Errachidia, Maroc

**Keywords:** Obésité, surpoids, enfants scolarisés, oasis de Tafilalet, Maroc, Obesity, overweight, school children, oasis of Tafilalet, Morocco

## Abstract

**Introduction:**

L’obésité représente un sérieux problème de santé publique qui a un impact direct sur la santé physique et mentale des individus. L’objectif du présent travail est de déterminer la prévalence de l’obésité et du surpoids en milieu scolaire urbain et rural, chez une population infantile oasienne.

**Méthodes:**

Nous avons entrepris une étude transversale descriptive au sein des établissements scolaires publics urbains et ruraux de l’oasis de Tafilalet. Nous avons recruté un échantillon représentatif de 3684 enfants scolarisés appartenant à 39 écoles publiques primaires.

**Résultats:**

La moyenne d’âge était de 9,81 ± 2,13 ans. L’échantillon total s’est réparti en 1794 garçons (48,70%) et 1890 filles (51,30%). Deux mille trois cent neuf appartenant à l’urbain (62,70%) et 1375 au rural (37,30%). Selon les références de l’organisation mondiale de la santé, notre étude a révélé un taux d’obésité de 1,9% et 10,8% pour le surpoids. L’obésité et le surpoids n’étaient pas significativement associés avec le sexe des enfants.

**Conclusion:**

La prévalence de l’obésité dans l’oasis de Tafilalet est inférieure aux données nationales et internationales, le mode de vie et les habitudes alimentaires de cette population semblent être un facteur protecteur contre l’obésité et le surpoids.

## Abstract (en)

**Introduction:**

Obesity is a serious public health problem having a direct impact on physical and psychological health of individuals. The purpose of this study was to determine the prevalence of obesity and overweight in a child population attending urban and rural schools in the oasis of Tafilalet.

**Methods:**

We conducted a cross-sectional descriptive study within the urban and rural public schools in of the oasis of Tafilalet. We recruited a representative sample of 3,684 children attending 39 public primary schools.

**Results:**

The average age of patients was 9.81 ± 2.13 years. The total sample was divided into 1,794 boys (48.70%) and 1,890 girls (51.30%). Two thousand three hundred and nine lived in an urban area (62.70%) and 1,375 in a rural area (37.30%). According to the World Health Organization References, our study showed a rate of obesity of 1.9% and of 10.8% for overweight. Overweight and obesity were not significantly associated with children sex.

**Conclusion:**

The prevalence of obesity in the oasis of Tafilalet is less than the national and international values; lifestyle and dietary habits of this population appear to be a protective factor against overweight and obesity.

## Introduction

L’obésité est un problème majeur de santé publique, non seulement dans les pays développés mais aussi les pays en voie de développement ne sont pas épargnés. Depuis quelques dizaines d’années, on relève une nette augmentation de la prévalence de l’obésité, en particulier l’obésité infantile. Selon l’organisation mondiale de la santé (OMS) le nombre de nourrissons et de jeunes enfants en surpoids ou obèses dans le monde s’est accru, passant de 32 millions en 1990 à 41 millions en 2016 [1]. L’obésité infantile est associée à un risque accru d’apparition des maladies non transmissibles telles que les maladies cardiovasculaires, le diabète, les troubles musculosquelettiques (TMS) et certains cancers, ainsi que les troubles psychosociaux. En outre, 44% de la charge du diabète, 23% de la charge des cardiopathies ischémiques et entre 7% et 41% de la charge de morbidité de certains cancers peuvent être attribués au surpoids et à l’obésité [2]. Les enfants en surpoids ou obèses risquent davantage de le rester à l’âge adulte [3]. Au Maroc, peu d’études ont été menées pour décrire la situation de l’obésité et du surpoids en milieu scolaire [4]. Aux zones oasiennes aucun travail n’est réalisé sur la prévalence de l’obésité et le surpoids chez les enfants scolarisés. C’est dans le souci de combler ce vide que nous avons entrepris ce travail.

## Méthodes

**Type de l’étude et population étudiée**: une enquête prospective, transversale par questionnaire assisté a été effectuée à partir du 5 mai 2015 jusqu’au 11 novembre 2017. Elle a concerné 39 écoles publiques primaires de l’oasis de Tafilalet. La taille de l’échantillon a atteint 3684 enfants, ce qui dépassait 1% de la population cible. Les deux sexes ont été inclus de manière aléatoire dans les zones urbaines et rurales.

**Mesures anthropométriques**: le poids corporel des enfants a été mesuré à l’aide d’un pèse-personne électronique de marque Seca avec précision de 0.1 kg. Lors de la prise des mesures anthropométriques, les chaussures sont retranchées ainsi que le poids estimé des vêtements. Les sujets restent immobiles jusqu’à la stabilisation de la mesure [5]. La balance a été quotidiennement calibrée. Le poids est par convention exprimé en kilogramme (kg). La taille en centimètre (cm) a été mesurée en position debout, décoiffé, pieds joints et nus avec toise graduée permettant d’apprécier le dixième de centimètre [6].

**Evaluation de la corpulence**: l’indice de masse corporelle (IMC) pour l’âge a été calculé pour tous les enfants sous forme des z score. On utilisait le logiciel WHO anthroplus recommandé par l’OMS. On s’est basé sur les nouvelles normes de croissance de l’OMS pour évaluer le taux d’obésité et du surpoids chez les enfants [7]. 1) Surpoids: >+1 z (équivalent à IMC de 25 kg/m^2^ à 19 ans). 2) Obésité: >+2 z (équivalent à IMC de 30 kg/m^2^ à 19 ans).

**Aspect éthique**: avant d’entamer l’enquête auprès des enfants scolarisés, des règles éthiques et déontologiques ont été prises en considérations. Les écrits ministériels pour but d’avoir les autorisations auprès de différentes délégations provinciales de l’éducation nationale ainsi que le consentement des parents ou tuteurs d’enfants. Nous avons fait des pré-visites aux écoles primaires choisies et donné les autorisations aux directeurs des établissements scolaires où les enquêtes auront lieu. Nous avons fait une présentation de nos objectifs afin de préparer l’environnement approprié et communiqué toute information utile qui va garantir un bon déroulement des enquêtes. Les questionnaires ont été anonymes durant toute l’enquête.

**Critères d’inclusion et d’exclusion**: durant la période de l’étude, les élèves de la 1^ère^ à la 6^ème^ année de primaire dont les parents ont accepté la participation ont été inclus dans ce travail. Nous n’avons exclus de l’étude que les élèves qui n’ont pas accepté de participer ou qui étaient absents au moment de l’enquête.

**Analyse statistique**: les différentes données obtenues ont été saisies et codées en utilisant le tableur Excel (version 2013), l’analyse a été effectuée à l’aide d’un logiciel statistique adéquat. Les données ont été représentées en moyenne ± écart-type (ET) et effectifs. Les analyses corrélationnelles ont été faites selon les types variables (quantitatif, qualitatif, nominal, ordinal), le test d’ANOVA à un facteur a été utilisé. Les valeurs de p<0,05 ont été considérées comme significatives.

## Résultats

Sur les 3684 enfants, 48,70% ont été des garçons et 51,30% ont été des filles avec un sex-ratio G/F de 0,95. L’âge moyen a été 9,82 ± 2,13 ans pour les garçons et 9,80 ± 2,14 pour les filles. Le poids moyen était 29,30 kg ± 8,12; la médiane est de 28,00 kg et le mode 25,00 kg. La taille moyenne était à 132,78 cm, la médiane à 133,00 cm et le mode à 132,00 cm. La moyenne de l’IMC pour âge est de -0.27 ± 1.18 chez les filles et -0,32 ± 1,23 chez les garçons. L’analyse statistique n’a révélé aucune signification entre les caractéristiques anthropométriques et le sexe des enfants (Tableau 1). Après l’utilisation de la classification de l’OMS pour l’IMC, notre étude a révélé un taux d’obésité de 1,9% et 10,8% pour le surpoids formant ainsi un total de 12,9% dans les deux milieux urbain et rural (Tableau 2). La prévalence de l’obésité et du surpoids par rapport au milieu de résidence (urbain ou rural), sexe et l’âge est montrée dans le Tableau 3. Des taux d’obésité et de surpoids ont été constatés chez les deux sexes et dans les deux milieux. La prévalence globale de l’obésité obtenue dans notre étude était 39/1890(2,06%) pour les filles et 31/1794(1,73%) pour les garçons. Concernant le surpoids, les deux sexes sont exposés d’une manière quasi équitable soit 5,27%(194/3684) pour les garçons et 5,56%(205/3684) pour les filles, la corrélation entre la corpulence et le sexe n’était pas statistiquement significative (p=0,184). La répartition de la prévalence de l’obésité et du surpoids selon l’âge des enfants, chez les deux sexes appartenant aux zones urbaines et rurales montre que globalement les enfants âgés de 11 ans prédominent (21,53%) mais la tranche d’âge de [7-10] ans est touchée d’une manière égale (13,86%) avec une légère augmentation de l’ordre 0,64% qui a été notée chez les enfant âgés de 10 ans. Pour tous les âges considérés en fonction du sexe, la prévalence de l’obésité et du surpoids est plus élevée chez les enfants vivant en urbain que ceux qui vivent en zone rurale. En effet le taux global de l’obésité en milieu urbain (11,73%, 55/469) est trois fois plus important que celui trouvé en zone rurale (3,20%, 15/469). Le surpoids en milieu urbain était quasiment égal chez les deux sexes, soit un taux de 52,29%(160/306), contre 47,71%(146/306), de même pour l’obésité.

**Tableau 1:** Caractéristiques anthropométriques des enfants scolarisés

Caractéristiques anthropometriques	Filles(n=1890) M(ET)	Garcons(1794) M(ET)	p
Age(an)	9,80(2,14)	9,82(2,13)	0,788
Poids(kg)	29,53(8,50)	29,06(7,69)	0,079
Taille(cm)	132,82(11,53)	132,72(11,48)	0,792
IMC(z score)	-0.27(1,18)	-0,32(1,23)	0,184

M: moyenne; ET: écart type

**Tableau 2:** Pourcentage de l’obésité et du surpoids chez les enfants scolarisés

Corpulence	N(%)
Surpoids	399(10,8)
Obésité	70(1,9)
Total	469(12,9)

N: effectif; %: pourcentage

**Tableau 3:** Surpoids et obésité en fonction des zones rurales et urbaines, le sexe et l’âge

Caractéristiques	Surpoids (%)	Obésité (%)	p
Sexe			0,227
Garçons	10,5	1,7	
Filles	11,1	2,0	
Age (an)			0,005
5-9	11,1	2,0	
10-12	11,1	1,8	
≥ 13	7,7	1,1	
Lieu de résidence			0,000
Urbain	13,2	2,3	
Rural	6,7	1,0	

## Discussion

L’objectif de cette étude était de déterminer la prévalence de l’obésité en milieu urbain et rural de l’oasis de Tafilalet au sud-est du Maroc. L’âge moyen était de 9,82 ± 2,13 ans. La taille de l’échantillon était de 3684 enfants scolarisés. La prévalence de l’obésité était de 1,9% et 10,8% pour le surpoids. Au niveau national très peu d’études ont été menées pour déterminer le taux de l’obésité et du surpoids. Un peu plus de trois % et démi d’obésité a été enregistrée à Rabat la capitale du Maroc, l’étude a été faite sur un échantillon de 1570 enfants âgés de 7 à 14 ans [4]. À Marrakech, 11% d’obésité qui a été révélée dans une étude qui a concerné 1418 enfants âgés de 8 à 15 ans selon la classification: « International de Obesity Task Force (IOTF, 2000)» [8]. Internationalement, 8,7% d’obésité en Tunisie, 23,10% en Algérie [9-10]. Plus de dix-neuf% en France auprès des enfants âgés de 12 ans. Face à ces différentes données, nous ne pouvons pas faire de comparaisons et tirer des conclusions pertinentes et puissantes car elles s’avèrent difficiles, voire impossibles en vue de l’hétérogénéité des méthodes utilisées à savoir, la diversité des références pour classer la corpulence des enfants, ajoutant la taille d’échantillonnage, les tranches d’âge concernées et les instruments de mesures. Mais il ressort de la présente étude que le taux d’obésité est inférieur aux valeurs nationales et d’autres internationales.

De nos résultats, il ressort que l’obésité en milieu scolaire urbain est plus importante que celle du rurale, ces résultats allant en concordance avec plusieurs études dans la littérature [11-14]. L’on pourrait expliquer par les changements des habitudes alimentaires et le style de vie par l’effet de l’urbanisation constituant ainsi un facteur de risque d’être atteint par la prise pondérale. Les différences de l’IMC par rapport au sexe n’étaient pas statistiquement significatives par comparaison aux autres travaux où la prédominance de l’obésité varie selon le sexe d’une manière significative [15-18]. Dans le présent travail le surpoids en milieu urbain était quasiment égal chez les deux sexes contrairement aux études qui révèlent des différences entre les sexes selon la variable de résidence urbaine ou rurale [19,20]. Dans notre étude, l’âge le plus touché est celui de 11 ans contrairement à une étude qui a été menée au Togo qui a révélé une prédominance en surpoids et obésité chez les enfants âgés de 15 et 16 ans, ceci semble être du fait de la représentativité des âges dans chaque étude ainsi que les spécificités des populations étudiées et le contexte environnemental [21]. D’autre part une étude métanalytique a reporté la tranche d’âge de 10 à 18 ans comme la plus touchée par l’obésité et le surpoids [22].

## Conclusion

Dans la présente étude le taux d’obésité est inférieur aux données nationales et internationales. Cet écosystème oasien bien particulier par ces habitudes alimentaires, son mode de vie et ces préparations culinaires à dominance végétale semble être un facteur protecteur contre l’obésité infantile.

### Etat des connaissances actuelles sur le sujet

- L’obésité touche les enfants de tout âge et les deux sexes;
- L’obésité concerne les enfants en milieu urbain plus qu’en milieu rural.

### Contribution de notre étude à la connaissance

- Les données sur l’obésité chez les enfants sont très rares en milieu oasien;
- Le taux d’obésité des enfants en milieu oasien est très faible en comparaison avec d’autres milieux d’étude.

## Remerciements

Nous tenons à remercier les établissements, leurs responsables et leurs élèves qui ont participé à cette étude.
